# A Rare Case of Testosterone-Producing Non-Seminoma Germ-Cell Testicular Cancer

**DOI:** 10.1210/jcemcr/luae244

**Published:** 2024-12-26

**Authors:** Sarah-Ålivia Mänd, Åke Sjöholm

**Affiliations:** Department of Internal Medicine, Gävle Hospital, SE-80324 Gävle, Sweden; Department of Internal Medicine, Division of Endocrinology and Diabetology, Gävle Hospital, University of Gävle, SE-80324 Gävle, Sweden

**Keywords:** non-seminoma germ-cell tumor, germ-cell tumor, testosterone, androgen

## Abstract

Androgen secretion by testicular germ-cell tumors (GCTs) appears to be markedly rare and likely underreported in the literature. This case study highlights a patient with such a rare tumor, underscoring a notable and yet easily avoidable diagnostic oversight in one of the most prevalent cancers among men. We advocate for increased vigilance and the inclusion of specific symptomatic screening for hyperandrogenism of select patients in existing guidelines and, where appropriate, the implementation of standardized hormonal laboratory analyses in both pre- and post-orchidectomy assessments. These measures could enhance the reporting of cases, standardize care, and improve understanding of the underlying mechanisms of these rare tumors. Finally, future studies should explore the implications of androgen secretion for the prognosis and treatment of GCTs.

## Introduction

Historically, characteristic hormonal secretion patterns have been associated with certain subtypes of testicular cancer (TC) [1]. In contrast to these established secretory patterns, a rare patient case with a non-seminoma mixed germ cell tumor (NSGCT) secreting androgen is presented below.

With a steadily increasing incidence over the past 4 decades, TC is the most commonly diagnosed solid-organ malignancy in males aged 15 to 44 years [2]. In 2020, more than 9300 deaths from TC were estimated worldwide [3]. While rare subtypes like Leydig- and Sertoli-cell tumors exist, germ-cell tumors (GCTs) account for about 95% of all TC [4]. The 2 main types are seminomas and NSGCTs, which include 4 histological subtypes: yolk sac tumors, embryonal cell carcinomas, choriocarcinomas, and teratomas [5]. Mixed NSGCTs are common, often more aggressive, and may include seminomatous elements. GCTs are further characterized by varying serum tumor biomarkers, such as alpha-fetoprotein (AFP), human chorionic gonadotropin (hCG), and lactate dehydrogenase (LDH) [6].

Androgen and estrogen secretion is typically associated with Leydig cell tumors rather than GCTs; however, a few rare cases of GCTs secreting androgens have been reported [2, 7, 8]. Based on the level of androgen excess, symptoms can include acne, oily skin, increased muscle mass, excessive body or facial hair, thinning scalp hair, mood changes, and heightened libido [9, 10]. During puberty, androgens and estrogens are critical for the development of the male reproductive system, and disruptions during this period have been linked to an increased risk of TC [11]. In adulthood, the relationship between hormone levels and TC risk is less researched and more complex [11].

Although managing TC may appear straightforward for clinicians due to established guidelines and improving treatment options, the abnormal hormonal patterns observed in the patient discussed below reveal a critical gap in current diagnostic approaches and suggest the need to incorporate hormone level data into existing diagnostic guidelines of GCT.

## Case Presentation

A 31-year-old, ethnically Syrian male with no previous medical history sought care due to several months of unilateral swelling and size increase of the right testicle. The patient did not report any symptoms suggestive of hyperandrogenism. The patient's risk factors for cancer were limited to active smoking (equivalent of 6.5 pack-years). His family history included no cases of cancer among first-degree relatives. However, a second cousin had been diagnosed with an unspecified type of TC at the age of 5.

## Diagnostic Assessment

**Figure 1.**
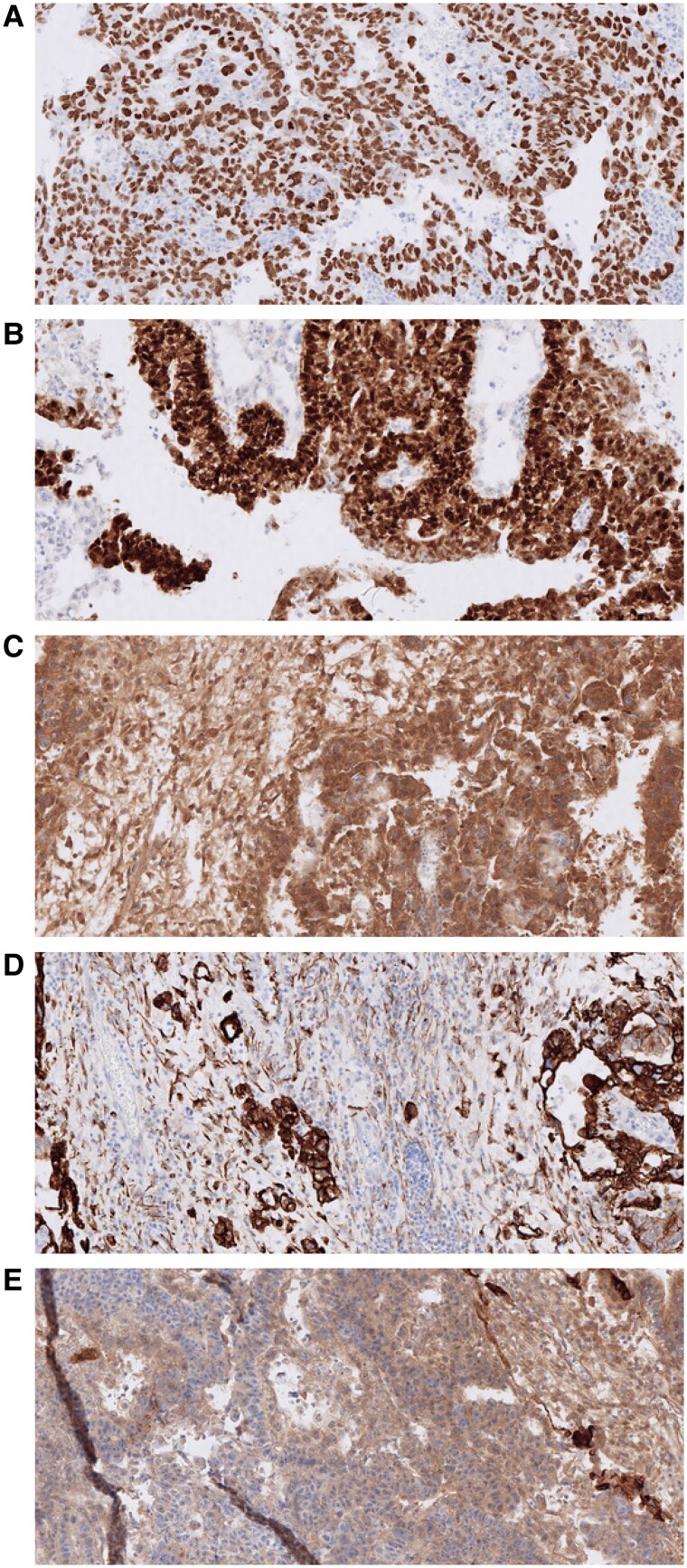
Histological analysis of the right testicle, revealing a non-seminomatous mixed germ cell tumor, comprising components of embryonal carcinoma, yolk sac tumor, and choriocarcinoma. Immunohistochemical staining was performed to identify specific tumor markers: A) Sal-like protein 4 (SALL4), B) Octamer-binding transcription factor 4 (Oct-4), C) Human chorionic gonadotropin (hCG), D) Cytokeratin pan, and E) Alpha-fetoprotein (AFP).

No palpable lumps in the scrotum were noted upon physical examination. However, ultrasound analysis revealed a right-sided testis tumor of 3 × 4 cm. As shown in Table 1, preoperative tumor markers and hormone levels were substantially elevated; specifically, the testosterone level was clearly supraphysiological. The patient denied any usage of anabolic steroids. The decision to assess testosterone levels was made by the attending urologist; however, specific clinical reasoning for this choice was not documented in the medical records and attempts to obtain clarification were unsuccessful. A preoperative computed tomography (CT) scan disclosed several paraaortic lymph nodes measuring < 8 mm. Following cryopreservation of sperm, a unilateral right-sided orchidectomy and a contralateral testicular biopsy were performed. Histological analysis showed a NSGCT with components of embryonal carcinoma, yolk sac tumor, and choriocarcinoma (Fig. 1). It was radically removed, showing growth into rete testis and lymphatic vessel invasion. The contralateral testis biopsy was normal.

**Table 1.** Pre- and postoperative hormonal laboratory analyses

Biomarker	Preoperative	Postoperative 1 week	Postoperative 8 weeks	Postoperative 10 months	Normal range
Alpha-fetoprotein (αFP)	221 kIU/L	102 kIU/L	7.3 kIU/L	5.4 kIU/L	0-5.8 kIU/L
Human chorionic gonadotropin (hCG)	12 kIU/L	1.3 kIU/L	<0.2 kIU/L	<0.6 kIU/L	0-2.0 kIU/L
Lactate dehydrogenase (LDH)	3.9 μkat/L (234 U/L)	2.6 µkat/L (156 U/L)	2.5 µkat/L (150 U/L)	3.4 µkat/L (204 U/L)	1.8-3.4 µkat/L (108-204 U/L)
Testosterone	36 nmol/L (1037 ng/dL)	2.3 nmol/L (66 ng/dL)	8.5 nmol/L (245 ng/dL)	13.5 nmol/L (389 ng/dL)	8.6-29.0 nmol/L (248-836 ng/dL)
Follicle-stimulating hormone (FSH)	<0.1 IU/L	1 IU/L	2 IU/L	2.9 IU/L	1.5-12 IU/L
Luteinizing hormone (LH)	<0.1 IU/L	1 IU/L	2.5 IU/L	4.2 IU/L	1.7-8.6 IU/L
Sex hormone-binding globulin (SHBG)	25 nmol/L (2.81 µg/mL)	ND*^a^*	28 nmol/L (3.15 µg/mL)	26 nmol/L (2.92 µg/mL)	18-54 nmol/L (2.02-6.07 µg/L)

Table shows serum levels of various tumor biomarkers and hormones before and after surgical removal of the testicular tumor in Système International (SI) and conventional units in parenthesis. Normal ranges are based on the laboratory references provided by Gävle Hospital's chemical laboratory.

^*a*^No data.

## Treatment

In accordance with the national Swedish guidelines for TC [12], our patient was promptly referred post-surgery to an oncology clinic for specialized care. The patient received a single dose of adjuvant chemotherapy (bleomycin, etoposide and platinol/cisplatin [BEP]).

## Outcome and Follow-Up

Postoperative tumor markers and hormonal levels showed clear decreases (Table 1). As expected, after removal of the androgen-producing tumor, the suppressed gonadotrophin levels were normalized. Postoperative hematocrit was within the normal range; however, it was not measured preoperatively. A 3-month postoperative follow-up CT scan showed spontaneous regression of the paraaortic lymph nodes, measuring 5 mm. No distant metastases were found. As of November 2024, the patient remains in remission.

## Discussion

Testicular nodules accompanied by symptomatic hyperandrogenism often lead to suspicion of Leydig cell tumors, as such tumors are commonly associated with the oversecretion of androgens and estrogens [13]. Our current report illustrates an unusual case of a NSGCT with components of embryonal carcinoma, yolk sac tumor, and choriocarcinoma with an androgenic oversecretion pattern. A comprehensive literature research yielded only 3 prior case reports with confirmed GCT androgen- or estrogen oversecretion [2, 7, 8].

The role of androgen secretion in GCT behavior remains poorly understood. In contrast, androgens are well-known drivers of prostate cancer, where androgen receptor (AR) signaling promotes tumor progression. This makes androgen deprivation therapy a standard prostate cancer treatment, unlike in TC [14, 15]. Interestingly, research by Weijun Jiang et al suggests that genetic polymorphisms in the AR may also modulate the risk and behavior of specific TC subtypes. It remains unclear, however, whether these genetic variants directly impact AR function or influence androgen-tumor interactions [16]. In our study, elevated androgen levels correlated with tumor activity, raising questions as to whether androgens actively promote tumor progression or merely reflect tumor presence. This uncertainty highlights the need for further research into the relationship between AR, androgen secretion, and GCT behavior.

The limited understanding of hormonal secretion in TC remains apparent; nevertheless 5-year survival rates in the United Kingdom have surged from 69% to 98% between the 1970s and 2010s, primarily due to advancements in early detection, improved treatment strategies, and increased awareness [17]. Clinicians worldwide benefit from various flow charts that guide TC diagnosis and treatment [12, 18-20]. However, many of these charts may not adequately emphasize the importance of biomarker- and hormone levels, such as testosterone, estrogens, follicle-stimulating hormone (FSH), luteinizing hormone (LH), and sex hormone binding globulin (SHBG).

The European Association of Urology (EAU) operates internationally, providing standardized diagnostic guidelines for Standardized Course of Care for several cancer forms [21]. The EAU Standardized Course of Care for TC offers a comprehensive diagnostic protocol covering symptoms, palpable testicular nodules, ultrasound examinations, tumor markers, CT scans, and more. Clinicians are instructed to measure tumor markers, including serum hCG, LDH, AFP pre- and postoperatively. The guidelines also mention “other tumor markers” including micro RNAs. There is no mention of testosterone, estrogen, FSH, LH or SHBG. Similar fast-track schemes are available from national institutions in the United Kingdom, the United States, and other countries. Among these, some suggest a widened “hormonal status” [22, 23]; however, there exists a lot of variability between different guidelines. Some guidelines suggest no clear hormonal status at all, apart from established tumor markers [12, 15, 24, 25].

The lack of standardized guidelines for hormonal testing in TC patients creates a significant barrier to fully understanding androgen-secreting GCT and their clinical implications. For patients showing symptoms of hyperandrogenism, comprehensive hormonal assessments are essential, as they help distinguish active tumor characteristics, confirm androgen excess from the tumor following removal, and may act as an indicator of relapse later in life. Since hyperandrogenism is not commonly associated with most TC subtypes, many clinicians may overlook these rare tumors and their subtle signs. This underscores the need for greater awareness and standardized guidance in clinical practice. Incorporating diagnostic flowcharts that highlight key symptoms of androgen excess could aid clinicians in recognizing hyperandrogenic stigmata. Such guidelines should encourage prompt standardized hormonal testing if symptoms are present and recommend structured follow-up if imbalances are detected. Additionally, clinicians should be mindful that hyperandrogenic symptoms, even in the absence of testicular complaints, warrant a thorough testicular examination to enable early TC detection.

An important dilemma is determining who should be screened. Currently, screening all TC patients for hormonal levels remains impractical due to the economic burden and the uncertain influence of androgen levels on prognosis. Moreover, treatment protocols typically remain unchanged whether or not androgen oversecretion is confirmed, as surgical removal of localized tumors generally restores hormonal balance. However, even if treatment remains unchanged, omitting pre- and postorchidectomy hormonal analyses in these patients may compromise the ability to assess treatment effects and relapse. Additionally, our case, alongside another patient presenting only with subclinical gynecomastia [2], highlights that some patients with dysregulated androgen levels may be at risk of underdiagnosis when only symptomatic individuals are screened. However, identifying asymptomatic patients would require implementing routine hormonal screening for all TC cases, a strategy that can only be justified through further research to establish the relevance of hormonal secretion in prognosis and treatment outcomes.

Moreover, even when screened, it is essential that hormonal assessments are conducted in a standardized manner. The inconsistent assessment of these hormones impairs the accurate estimation of the prevalence of these rare tumors, potentially contributing to the limited number of reported cases. This scarcity makes it difficult to discern whether their rarity is due to underdiagnosis or inherent biological factors. A brief review of hormones in published cases of androgen-secreting GCTs, including ours, underscores the lack of consistency: all studies tested for what could be called “hormonal status” [2, 7, 8]; however, only 1 out of 4 provides comprehensive data including FSH, LH, free and total testosterone, estrogen, SHBG, AFP, hCG, and LDH pre- and postoperatively [7]. In our case, estrogen, along with additional relevant tests—such as inhibin B and hematocrit—were missing preoperatively. This highlights the current absence of a standardized approach to assessing hormonal markers for androgen-secreting GCTs, even in patients deemed suitable for testing. Implementing a standardized panel of hormonal tests for future cases would help ensure consistency and facilitate meaningful comparisons across studies.

Another important gain of assessing hormone levels is insight into the functioning of these tumors. Due to limited study cases, the mechanisms of androgen and estrogen oversecretion in GCTs remain largely elusive. Hooman et al reported 2 cases of androgen-producing GCTs, suggesting that tumors might synthesize and release testosterone and estradiol, or that hCG—due to its structural similarity to LH—could stimulate Leydig cells to produce androgens [7]. In our patient's case, both preoperative hCG and testosterone levels were elevated, supporting these hypotheses. Rieu et al further demonstrated a positive correlation between hCG levels (5 to 3500 IU/L) and testosterone, while levels exceeding 3500 IU/L showed a negative correlation with serum testosterone. These findings emphasize that while orchidectomy may cure patients without hormonal assessment, evaluating hormone levels is critical for understanding the underlying mechanisms of GCTs.

In summary, current data lacks a clear understanding of the interplay between androgen secretion and GCTs. Collecting more data is essential to enhance our understanding of these tumors. To achieve this, existing guidelines should prompt clinicians to look for hyperandrogenic stigmata and, when present, recommend standardized hormonal screening. This approach would help identify more cases, ensure their comparability, determine the true prevalence of androgen-secreting GCTs, and clarify the potential effects of androgens and other hormones on prognosis and treatment.

## Learning Points

- This paper sheds light on a rare testicular tumor, with only a few prior cases documented, and highlights a significant diagnostic gap in one of the most common cancers affecting men.
- We advocate for the inclusion of specific symptomatic screening for hyperandrogenism into existing guidelines, and when appropriate on a case-by-case basis, hormonal laboratory analyses—such as FSH, LH, free/total testosterone, estrogen, SHBG, AFP, hCG, and LDH—in both pre- and postorchidectomy assessments.
- Such measures may contribute to (i) an increase in reported cases of androgen-secreting GCTs; (ii) standardized care and enhanced patient safety in managing testicular cancer; (iii) the generation of new data to advance research into the underlying mechanisms of androgen-secreting GCTs; and (iv) possibly also help identify relapse cases.

## Contributors

Both S-Å.M. and Å.S. made individual contributions to authorship and reviewed and approved the final draft.

## Data Availability

Restrictions apply to the availability of some or all data generated or analyzed during this study to preserve patient confidentiality or because they were used under license. The corresponding author will on request detail the restrictions and any conditions under which access to some data may be provided.

## Abbreviations

AFP: alpha-fetoprotein
AR: androgen receptor
CT: computed tomography
FSH: follicle-stimulating hormone
GCT: germ-cell tumor
hCG: human chorionic gonadotropin
LDH: lactate dehydrogenase
LH: luteinizing hormone
NSGCT: non-seminoma mixed germ cell tumor
SHBG: sex hormone binding globulin
TC: testicular cancer
